# The Neural Mechanisms Underlying the Acute Effect of Cigarette Smoking on Chronic Smokers

**DOI:** 10.1371/journal.pone.0102828

**Published:** 2014-07-22

**Authors:** Kangcheng Wang, Junyi Yang, Songyan Zhang, Dongtao Wei, Xin Hao, Shen Tu, Jiang Qiu

**Affiliations:** 1 Key Laboratory of Cognition and Personality (SWU), Ministry of Education, Chongqing, China; 2 School of Psychology, Southwest University, Chongqing, China; 3 Department of Psychology, Institute of Education, China West Normal University, Nanchong, Sichuang, China; Wake Forest School of Medicine, United States of America

## Abstract

Although previous research had related structural changes and impaired cognition to chronic cigarette smoking, recent neuroimaging studies have associated nicotine, which is a main chemical substance in cigarettes, with improvements in cognitive functions (e.g. improved attention performance). However, information about the alterations of whole-brain functional connectivity after acute cigarette smoking is limited. In this study, 22 smokers underwent resting-state functional magnetic resonance imaging (rs-fMRI) after abstaining from smoking for 12 hours (state of abstinence, SOA). Subsequently, the smokers were allowed to smoke two cigarettes (state of satisfaction, SOS) before they underwent a second rs-fMRI. Twenty non-smokers were also recruited to undergo rs-fMRI. In addition, high-resolution 3D T1-weighted images were acquired using the same magnetic resonance imaging(fMRI)scanner for all participants. The results showed that smokers had structural changes in insula, thalamus, medial frontal cortex and several regions of the default mode network (DMN) compared with non-smokers. Voxel-wise group comparisons of newly developed global brain connectivity (GBC) showed that smokers in the SOA condition had higher GBC in the insula and superior frontal gyrus compared with non-smokers. However, smokers in the SOS condition demonstrated significantly lower GBC in several regions of the DMN, as compared with smokers in the SOA condition. These results suggest that structural integrity combined with dysfunction of the DMN might be involved in relapses after a short period of time among smokers.

## Introduction

Cigarette smoking is one of the leading causes of morbidity and mortality globally [1], [2]. According to one report, approximately 4.9 million people died around the world in 2007 as a result of smoking [3]. A great interest of researchers is assessing the influence of chronic cigarette smoking on the human brain. Chronic cigarette smoking has been associated with structural changes in several key brain regions, including the medial frontal cortex [4], thalamus [5], [6], insula [7], parietal cortex [8], anterior cingulate cortex, and middle cingulate cortex [9]. Consequently, smokers have several difficulties in completing cognitive tasks, such as working memory [10], [11], delayed reward [12], and cognitive control tasks [13].

A growing body of evidence suggests that several regions of the brain display structural changes as a result of chronic cigarette smoking. Gons et al. (2011) used diffusion tensor imaging (DTI) to show that a history of cigarette smoking could be associated with the reduced microstructural integrity of white matter (WM) [14]. Compared with nonsmokers, chronic cigarette smokers have higher fractional anisotropy (FA) in the bilateral superior longitudinal fasciculus, which is a major WM pathway of fronto-parietal tracts [8]. Smokers also have higher FA in the prefrontal WM, cingulum cortex, and genu corpus callosum than nonsmokers [15]. Other studies have found that smokers have smaller gray matter volumes (GMVs) in the thalamus, medial frontal cortex, cingulate cortex, and bilateral prefrontal cortex than non-smokers through voxel-based morphometry (VBM). The gray matter (GM) densities in the bilateral prefrontal cortex, orbitofrontal cortex, occipital lobe, and the temporal lobe were also found to decrease for smokers [4], [5], [16].

Aside from changes in the brain structures of smokers, the influence of nicotine on brain functions after acute smoking is also of interest. In an early functional magnetic resonance imaging (fMRI) study, Stein et al. (1998) found increased activation in the insula, frontal lobes, and amygdala after cumulative intravenous nicotine administration in cigarette smokers [17]. Another study used fMRI to investigate the acute effects of nicotine on smokers and reported an improvement in the performance of smokers at a visual attention task after nicotine administration due to increased activation in the insula, frontal gyrus, caudate, and thalamus [18]. Recently, several studies have examined the nicotine effect on large-scale brain networks. Hahn et al. (2007) used event-related fMRI to investigate the influence of nicotine on smokers' attention and found that nicotine induced deactivation in the default mode network (DMN) and improved attention performance in smokers [19]. Cole et al. (2010) investigated the effects of nicotine replacement on abstinent smokers through resting-state fMRI (rs-fMRI); the therapeutic effect of nicotine replacement on cognitive withdrawal symptoms was associated with an enhanced inverse coupling between the executive control network and DMN [20]. Another study which used rs-fMRI also showed that smokers exhibited reduced connectivity in DMN regions and increased activity in the network related to attention after nicotine administration [21]. These results showed that nicotine was associated with decreased activity in DMN regions and increased activity in the regions related to executive control and attention.

Nicotine, which is a main chemical substance in cigarettes, can alter neural activity [19], [21] by activating nicotinic cholinergic receptors [22]. However, information on the effects of acute cigarette smoking on neural circuits remains insufficient [22]. Increasing evidence has shown that distributed neural circuits in the brain exhibit spontaneous activity while people are at rest [23]. These slow frequency fluctuations in brain activity are temporally correlated within functionally related networks [24]. Such evidence provides an opportunity to investigate and characterize neural circuit abnormalities in smokers [25]. However, no study has investigated global functional connectivity patterns after acute cigarette smoking, although prior findings constitute important advances in our understanding of addiction to smoking. Such a global, data-driven approach is important to comprehensively examine the changes in global brain connectivity (GBC) after acute cigarette smoking. Thus, the present study applied a recently developed GBC method that could identify specific nodes or hubs influenced by smoking. Moreover, whether the regions influenced by acute cigarette smoking are related to structural change remains unknown. Although previous studies have used VBM based on the analysis of regions of interest (ROIs) to explore the changes caused by chronic cigarette smoking to some extent, this method excludes some key regions [5], [8]. Therefore, a whole-brain analysis without an ROI-based hypothesis could be conducted to comprehensively investigate the structural brain changes in smokers and their relation to acute cigarette smoking.

To examine the effect of acute cigarette smoking on brain function and its relation to the regions that show structural changes, this study compared (1) the GBC of smokers when they abstained from smoking for 12 hours (state of abstinence, SOA) and after acute cigarette smoking (state of satisfaction, SOS) and (2) the GMV between smokers and non-smokers. On the basis of previous studies, we developed two a priori hypotheses. First, we hypothesized that smokers would display structural changes in prefrontal cortex and brain regions in DMN. Then, we hypothesized that the regional GBC of the DMN would be suppressed after acute cigarette smoking (i.e. in the SOS condition). Lastly, the regions of GBC changes caused by acute cigarette smoking would be associated with structural changes in chronic smokers, which would be expressed by increased or decreased GMV.

## Materials and Methods

### Participants

A total of 42 participants (22 male smokers and 20 male nonsmokers) aged 19 to 28 years-old were recruited through advertisements. All the participants recruited were undergraduates or graduates of Southwest University (Chongqing, China). After registration, potential participants were screened using semi-structured interview to assess their psychiatric condition, medication use, medical condition, history of substance use, history of claustrophobia, and the presence of metal implants in their body. Cigarette smokers who smoked at least eight cigarettes per day without a period of abstinence longer than a week in the past year and met the DSM-IV criteria for nicotine dependence were considered suitable for this study. All the non-smokers in this study had no history of smoking in any case. All the participants were right-handed native Chinese speakers.

The following exclusion criteria for smokers and non-smokers were used: acute physical illness, claustrophobia, history of head injury with skull fracture, presence of metal dentures, history of alcohol or drug abuse or dependence, history of central nervous system diseases or conditions, history of medical conditions with significant effects on the central nervous system, history of mental illness, and family history of psychopathic disorders.

Participants were fully informed of the measurement methods and magnetic resonance imaging (MRI) scanning. All participants gave their written informed consent. The local ethics committee of Southwest University approved this consent procedure and the experimental procedure, which were both in accordance with the standards of the Declaration of Helsinki. All participants were given appropriate compensation after completing this study.

### Experimental design and image acquisition

All cigarette smokers were asked to avoid smoking for 12 hours before MRI scanning; this state was defined as the “state of abstinence” (SOA). To measure the extent of craving, each smoker was asked to fill in the 10-item Questionnaire of Smoking Urges (QSU-brief) [26], [27] 10 mins before the first scanning. After scanning, the smokers were allowed to smoke two cigarettes; these smokers were in the “state of satisfaction” (SOS). The QSU-brief was again filled in by each smoker after the second scanning. Functional data of three of the smokers were removed because they had overly large head motions.

All MRI scans were obtained using a 3.0 T MRI scanner (Siemens, Erlangen, Germany). Participants were instructed to relax with their eyes closed, remain awake, lie still, and not to think of anything during rs-fMRI scanning. Participants were positioned carefully in the coil with a comfortable support and fitted with soft earplugs to limit the effects of noise on brain activity. After scanning, all participants were requested to confirm that they had not fallen asleep.

High-resolution T1-weighted anatomical images were acquired using a 3D magnetization-prepared rapid gradient-echo sequence in axial orientation (repetition time [TR]  = 1900 ms; echo time [TE]  = 2.52 ms; flip angle  = 9°; field of view [FOV]  =  256×256 mm^2^; matrix  =  256×256; voxel size  =  1×1×1 mm^3^).

T2-weighted fMRI images were acquired through a gradient-echo echo-planar pulse imaging sequence (TR  =  2000 ms; TE  =  30 ms; flip angle  =  90°; number of slices  =  32; slice thickness  =  3.0 mm; slice gap  =  1.0 mm; FOV  =  220×220 mm^2^; matrix  =  64×64; voxel size  =  3.4×3.4×4 mm^3^). All images were aligned along the anterior commissure–posterior commissure (AC–PC) line. A total of 242 volumes were acquired for each participant.

### VBM analysis

VBM is an automatic procedure that can differentiate GMV. VBM was performed using SPM 8 software (Welcome Department of Imaging Neuroscience, London) running on Matlab (version 7.10.0; Math-Works, Natick, MA, USA). All T1-weighted structural images were manually co-registered to the AC–PC line with the standard T1-weighted template provided by SPM 8 for better registration. The co-registered images from each participant were segmented into GM, WM, and cerebrospinal fluid (CSF) regions using the unified segmentation procedure. A diffeomorphic nonlinear registration algorithm named DARTEL (diffeomorphic anatomical registration through exponentiated lie algebra), was used to conduct a special normalization that involved the following steps. First, a specific template was computed using the average tissue probability maps from all the participants, and each participant's maps were warped into the specific template. This procedure was repeated until the best study-specific template was generated to improve the alignment and achieve a more accurate inter-participant registration. Second, further modulation was conducted to preserve the volume of GM/WM. This step involves multiplying the spatially normalized GM/WM by its relative volume [28]. Inference was made on the measures of volume rather than tissue concentration (density) when using modulated images for performing subsequent group comparisons. Finally, a 10 mm full-width at half-maximum (FWHM) Gaussian kernel [29], [30] was applied to smooth the modulated GM/WM images.

Based on the general linear model, voxel-based comparisons of GMV were performed between groups of smokers and nonsmokers using two-sample *t*-test. Age, education, and the whole brain volume (GMV and WM volume) were used as covariates to control for possible confounding variables in the whole-brain analysis. The significance of group differences was set at a 0.05 significance level (with a combined height threshold of p<0.001 and a minimum cluster size of 41 voxels) using the AlphaSim criterion as the threshold to correct for multiple comparisons. In addition, we investigated the relationship between changed regions and smoking history. We performed an exploratory correlation analysis between the GMV of changed regions and the smoking history of each smoker. First, we created five ROI volumes based on a threshold (at p = 0.05, AlphaSim corrected) significant peak of GMV to compare smokers and nonsmokers. We then extracted the GMV of each ROI for each smoker and correlated this value with years of smoking.

### GBC analysis

Pre-processing of all functional images was conducted using SPM 8. First, 10 volumes were removed to ensure steady-state longitudinal magnetization before slice timing and realignment were conducted. Data from three smokers and one nonsmoker were excluded because their translation or rotation exceeded ±2.0 mm or ±2.0°. Second, the realigned images were spatially normalized using an echo-planar imaging template and resampled into 3 mm cubic voxels. Third, a 6 mm FWHM Gaussian blur was used to smooth each 3D volume and decrease the spatial frequency noise. Finally, band-pass filtering from 0.01 Hz to 0.08 Hz was conducted, and several sources of nuisance covariates were regressed, namely, six head motion parameters, the global mean signal, the WM signal, and CSF.

GBC was calculated as previously described [31]–[34]. The connectivity between each voxel in the whole brain was estimated. The time course of each voxel from each participant was correlated with every other voxel. Thus, a matrix of Pearson's correlation coefficients was obtained. Subsequently, the number of voxel connections for each voxel was counted with a threshold of *r*>0.25 to compute the GBC map [31]. The vertex degree was calculated as the number of adjacent links using an undirected and weighted adjacency matrix. The map was finally transformed to Fisher *Z* values, so that maps across participants could be averaged and compared.

One-sample *t*-tests were performed for nonsmokers, smokers in the SOA condition, and smokers in the SOS condition to identify voxels with significantly higher connectivity. Significance threshold was set to p<0.01 (with a combined height threshold of p<0.01 and a minimum cluster size of 40 voxels) using the AlphaSim criterion.

A two-sample *t*-test was also performed to compare the *Z* value maps of SOA smokers and nonsmokers for identifying the GBC of smokers in the SOA condition. To determine GBC changes after acute cigarette smoking, a paired *t*-test was performed to compare the *Z* value maps of smokers in the SOA and SOS conditions. Moreover, a two-sample *t*-test was conducted to compare smokers in the SOS condition and nonsmokers to further investigate the influence of acute cigarette smoking. The *t*-map was set to a threshold of p<0.01 using the AlphaSim correction. Group comparisons were restricted to voxels with significant comparison maps of smokers or nonsmokers using an explicit mask from the union set of the one-sample *t*-test results.

## Results

### Participants

The participants' demographic and characteristic data are shown in Table 1. The smokers were matched with the nonsmokers by age (*T*-score  = 1.08, p>0.05) and years of education (*T*-score  = 0.13, p>0.05). The smokers had an average of 4.95 years of smoking and consumed an average of 11.90 cigarettes per day (Table 1). The mean (SD) score of pack-years [35] was 3.10±2.63. The mean (SD) craving scores, which were measured by the QSU-brief for smokers in the SOA and SOS conditions, were 44.42±10.61 and 32.32±10.24 respectively. The smokers indicated lower craving levels under the SOS condition (*T*-score  = 3.61, p = 0.002).

**Table 1:** Demographic and characteristic data of smoker and nonsmoker participants.

Characteristics	Smokers (n = 22, male)	Nonsmokers (n = 20, male)	t	*p*
Age (Mean ± SD years)	22.48±2.48	21.80±1.32	1.08	0.29
Education (Mean ± SD years)	15.14±1.83	15.20±1.19	0.13	0.90
Cigarettes (per day)	11.90±6.13	NA		
Years of smoking (Mean ± SD years)	4.95±2.27	NA		
Pack-years	3.10±2.63	NA		
QSU (SOA)*	44.42±10.61	NA		
QSU (SOS)*	32.32±10.24	NA		

* The number of participants for smoker's QSU scores was 19. The data of three smokers were removed as they were not included in further GBC analysis.

Abbreviation: QSU, Questionnaire of Smoking Urges; SOA, State of Abstinence; SOS, State of Satisfaction.

### VBM results

**Figure 1:**
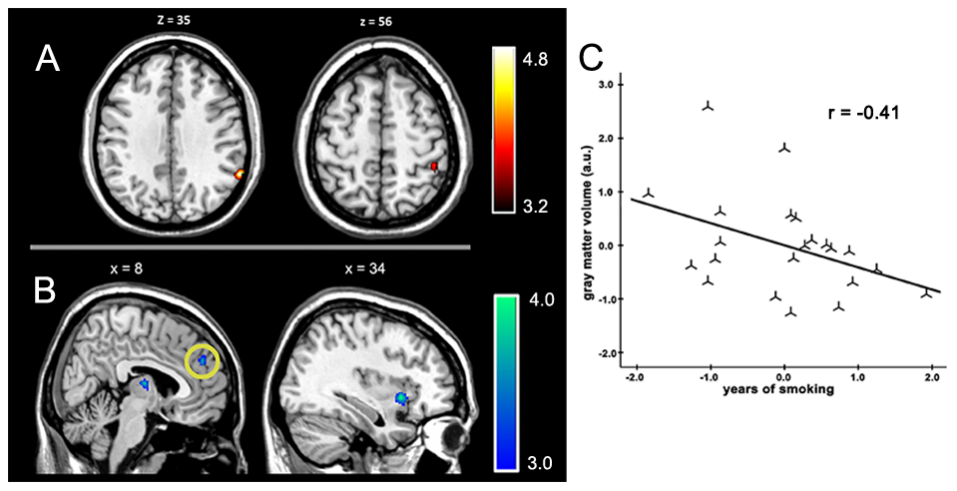
The effect of chronic cigarette smoking on GMV. Smokers showed higher GMV in the angular gyrus and inferior parietal lobule (A) and lower GMV in the insula, thalamus, and medial frontal cortex (B) compared with nonsmokers. GMV in the medial frontal cortex (B, region with yellow circle) was significantly negatively correlated with the history of smoking after controlling for age, education, and whole-brain volume (C). The results are shown with p<0.05 and corrected for multiple comparisons with the AlphaSim correction.

The two-sample *t-*test (p<0.05 with the AlphaSim criterion) revealed significant differences between the GMVs of smokers and non-smokers. Smokers had significantly more GMV in the right angular gyrus (*x*, *y*, *z* = 62, -50, 35; *T*-score  = 4.78) and inferior parietal lobule (*x*, *y*, *z* = 45, -38, 56; *T*-score  = 3.98) compared with non-smokers. Smokers' were found to have lower GMV in the right thalamus, right medial frontal gyrus, and right insula. The voxels of the peak GMV differences were in the right thalamus (*x*, *y*, *z* = 6, -11, 9; *T*-score  = 3.97), right medial frontal gyrus (*x*, *y*, *z* = 11, 48, 30; *T*-score  = 3.77), and right insula (*x*, *y*, *z* = 35, 14, -3; *T*-score  = 3.94) (for additional information, see Table 2 and Figure 1).

**Table 2:** Differences in gray matter volumes between smokers and nonsmokers.

Comparisons	R/L	Number of Voxels in Cluster	Regions	MNI coordinate			T score
				X	Y	Z	
smokers > nonsmokers	R	359	angular gyrus	61	−49	35	4.78
	R	52	inferior parietal lobule	45	−37	56	3.98
smokers < nonsmokers	R	133	Insula	34	13	−3	3.94
	R	47	thalamus	6	−10	9	3.97
	R	64	medial frontal cortex	10	48	30	3.77

The exploratory correlation analysis between the GMVs of ROIs and smoking history showed a negative correlation between years of smoking and volume of the right medial frontal gyrus (*r* = −0.491, p = 0.02). The correlation remained significant after controlling for age, education, and whole-brain volume (*r* = −0.413, p = 0.06; Figure 1).

### GBC results

**Figure 2:**
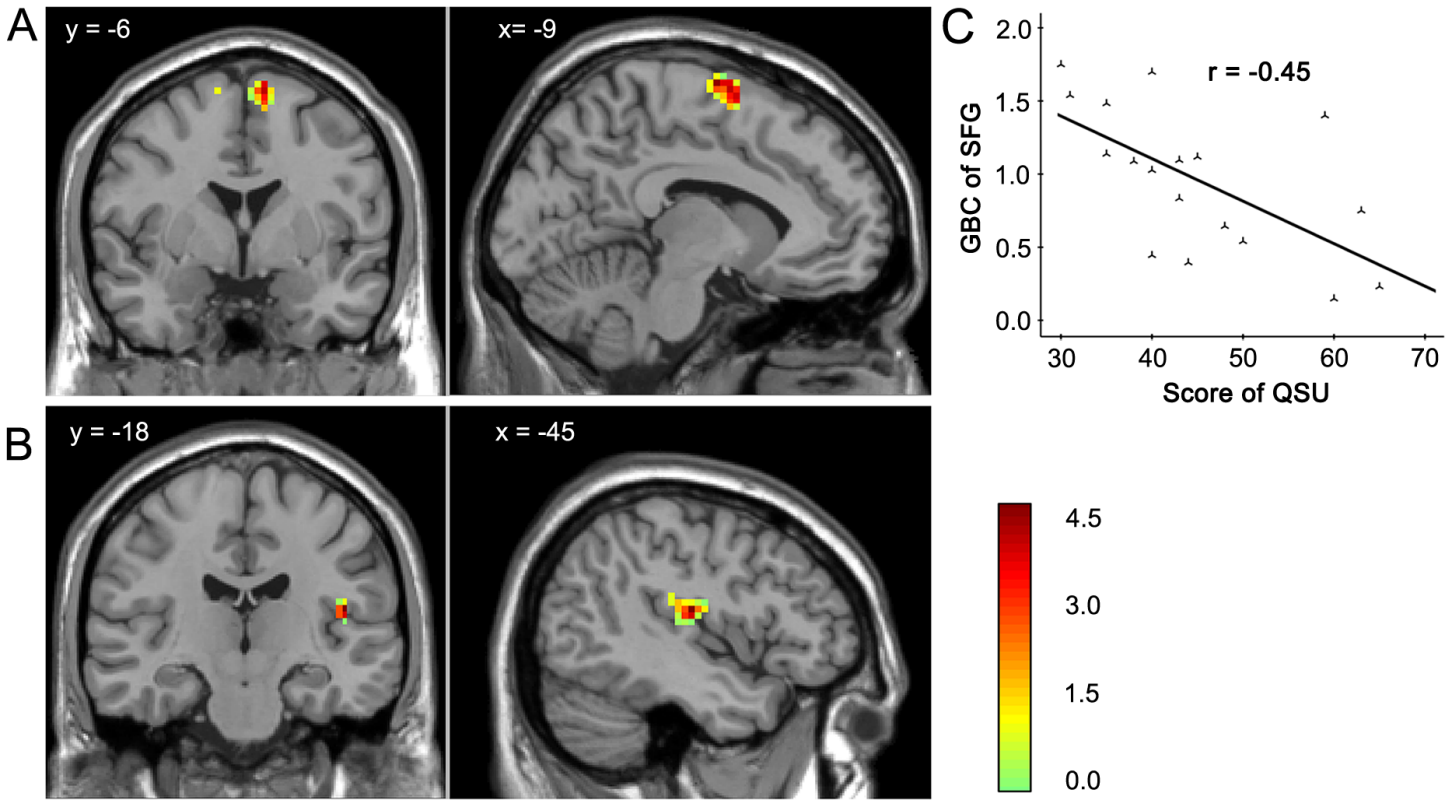
Regions of higher GBC in smokers under the SOA condition. Compared with that of nonsmokers, the GBC of smoker under the SOA condition was higher in the insula (A) and superior frontal gyrus (B). The GBC of the superior frontal gyrus was negatively correlated with the QUS-brief scores (C). The results are shown with p<0.01 and corrected for multiple comparisons with the AlphaSim correction.

The results of the two-sample *t*-test revealed higher GBC in smokers in the SOA condition than in nonsmokers in the insula (*T*-score  = 4.79; *x*, *y*, *z* = −45, −18, 15) and superior frontal gyrus (*T*-score  = 4.70; *x*, *y*, *z* = −9, −6, 72; Table 3 and Figure 2). The inclusion of age and education as covariates did not alter the results. The correlation analysis between the GBC of the insula and superior frontal gyrus and craving scores, which was measured by the QSU-brief, showed that craving scores were negatively correlated with the GBC of the superior frontal gyrus (*r* = −0.452, p = 0.05) but was uncorrelated with the GBC of the insula (*r* = 0.041, p = 0.868) in smokers in the SOA condition.

**Table 3:** Regions of higher global brain connectivity for smokers under the state of abstinence compared with nonsmokers.

Regions	R/L	Number of Voxels in Cluster	MNI Coordinates x, y, z	T score
Insula	L	45	−45, −18, 15	4.79
Superior frontal cortex	L	70	−9, −6, 72	4.70

**Figure 3:**
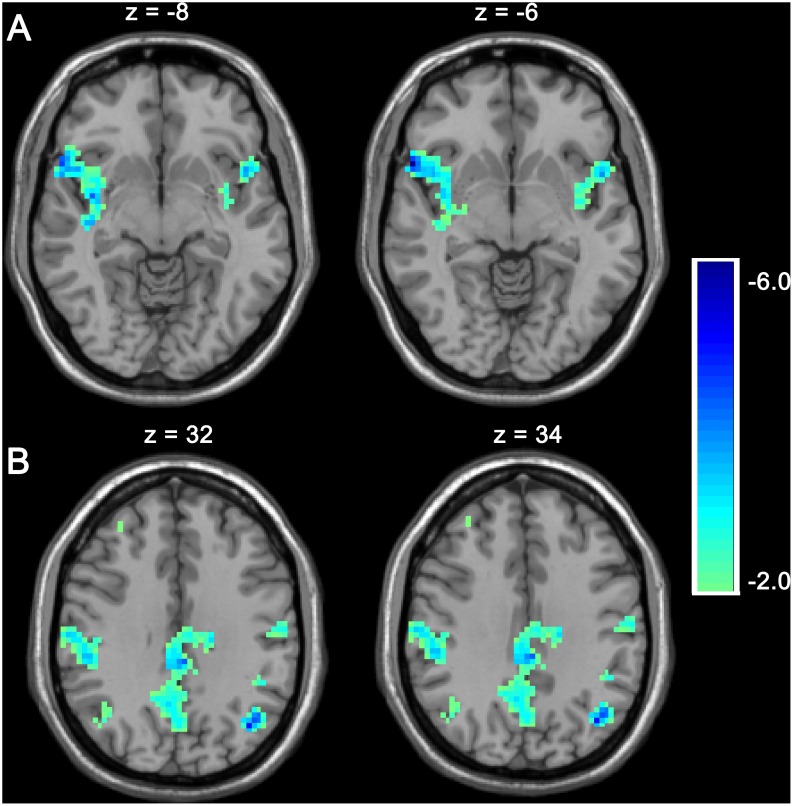
Regions of decreased GBC in smokers after acute cigarette smoking. Smokers showed decreased GBC the in bilateral insula (A) and regions of the DMN (B), including the middle frontal cortex, precuneus, bilateral angular gyrus, and bilateral inferior parietal lobule. The results are shown with p<0.01 and corrected for multiple comparisons with the AlphaSim correction.

The paired t-test between smokers in the SOS and SOA conditions showed that the former had decreased GBC in the bilateral insula and regions of the DMN, which included the precuneus, bilateral angular gyrus, and bilateral inferior parietal lobule (Figure 3). The two-sample t-test between smokers in the SOS condition and nonsmokers demonstrated that GBC in the intrinsically organized DMN was lower for smokers in the SOS condition. The affected brain regions were the middle frontal cortex, precuneus, bilateral angular gyrus, and bilateral inferior parietal lobule (Table 4). After correcting for age and education, it still reached the significant level.

**Table 4:** Regions of lower global brain connectivity for smokers under the state of satisfaction.

Regions	R/L	Number of Voxels in Cluster	MNI coordinates x, y, z	T score
Angular gyrus	R	86	45 −66 33	5.06
Angular gyrus	L	51	−48 −69 33	5.22
Middle frontal cortex	R	55	42 27 39	4.12
Inferior parietal lobule	R	98	57 −42 33	6.38
Inferior parietal lobule	L	56	−57 −45 39	4.15
Precuneus	R	77	3 −57 39	3.96

## Discussion

In the present study, we observed the structural changes among smokers in the insula, medial frontal gyrus, and several DMN regions, such as the angular gyrus and the inferior parietal lobule. In addition, under the SOA condition, smokers demonstrated higher GBC in the insula and superior frontal cortex. After acute cigarette smoking, however, smokers showed lower GBC in the DMN, including the middle frontal cortex, precuneus, bilateral angular gyrus, and bilateral inferior parietal lobule, which are a group of brain areas important in goal-directed cognitive performance [36]. These findings suggest that the structural changes in the DMN and the decreased global functional connectivity in the DMN of smokers after acute smoking might be involved in relapses.

Firstly, the present study showed that smokers demonstrated anatomical changes in the thalamus, medial frontal gyrus, and insula. These results are similar to those of previous studies that demonstrated brain structural changes caused by chronic cigarette smoking. Studies that performed VBM analyses have reported decreased GMV in the left thalamus and medial frontal cortex of smokers relative to those of control participants [4]. A lesion study found that smokers with a damaged insula were more likely to quit smoking [7]. In addition, Angelica (2014) found that cigarette exposure, dependence and craving was negatively correlated with insula [37]. These findings suggested that the insula could be a critical neural substrate of addiction to smoking. The present study also provided evidence of structural changes in the insula of smokers. The reason for such structural changes in smokers may be that chronic cigarette smoking may damage microvessels and influence the blood supply to the brain [38]. Smoking may also damage the neurons of the brain and lead to neuronal necrosis. As a result, chronic cigarette smoking may lead to brain structural changes.

Moreover, our results demonstrated that smokers under the SOA condition showed higher GBC in the insula and superior frontal gyrus than non-smokers. According to previous studies, the insula has the highest density of nicotinic acetylcholine receptors [39] and is a key neural structure for representing the interoceptive effects of addiction [40]. It plays a role in enabling a subjective experience about the body's primary interoceptive and exteroceptive information and emotional feeling states [41]. In the present study, after 12 hours of abstinence, smokers showed a strong urge for smoking. Thus, the high GBC in the SOA condition might reflect that the smoker's primary interoceptive and exteroceptive information and emotional information were integrated and believed to generate conscious awareness of feeling states of craving. The prefrontal cortex is the most commonly reported loci of activation related to the pathogenesis of craving. A previous study using the rTMS method showed that the superior frontal gyrus had excitatory and inhibitory influences on cravings and the modulations of reactivity to cravings [42]. Moreover, a DTI study [43] implied that the superior frontal gyrus could be a major cortical “hub” with extensive interconnections, as identified in neuroimaging studies of cue-elicited cravings [44]–[46]. The present study found that craving scores were negatively correlated with the GBC of the superior frontal gyrus, and provided further evidence of the role of the superior frontal gyrus in the inhibitory effect of cravings.

More interestingly, smokers under the SOS condition showed lower GBC in the middle frontal cortex, precuneus, bilateral angular gyrus, and bilateral inferior parietal lobule. This deactivated network overlaps the DMN. This finding is consistent with previous studies that demonstrated reduced BOLD-related activity in DMN regions after the administration of nicotine or nicotinic cholinergic agonists [19], [21], [47]–[49]. Previous studies have suggested that the degree of deactivation or suppression of the DMN is related to task demands [50]. This finding suggested a reallocation of resources away from the DMN toward the regions involved in task performance [21]. Many studies have also associated DMN deactivation with goal-directed cognitive processes, such as focused attention and working memory [51]–[55] and the enabling of systemizing and problem solving by insight [56]. Thus, the lower GBC in the DMN after acute cigarette smoking observed in the present study could contribute to smokers' decreased negative attentional bias and increase of good performance in some cognitive behaviors. Indeed, well-documented studies have demonstrated that smoking could enhance the performance of cognitive behaviors, including attention, information processing, and memory [57].

Moreover, our results demonstrate that GMV-based regions of structural change partially overlapped with the GBC results. However, the relationship between structure and function remains unclear [58]. The most obvious explanation is that the structural changes caused by chronic cigarette smoking could lead to functional abnormalities, as demonstrated by the structural changes in the insula and frontal gyrus that led to higher GBCs of smokers in the SOA condition. This result was in line with a previous study of the influence of structural changes on brain function [58], [59]. For instance, callosal agenesis decreases the inter-hemispheric functional connectivity during the resting state [60], [61]. In major depressive disorders, structural abnormalities of the uncinate fasciculus are associated with increased functional connectivity between the subgenual anterior cingulate cortex and the medial temporal lobe, which are concomitant with the severity of depressive symptoms. Our study provided additional evidence for this structure–function relationship. For the DMN, structural changes and decreased GBC after acute cigarette smoking were found in our results. They might be involved in the relapse of smokers. Structural changes caused by chronic cigarette smoking can lead to brain functional abnormalities and poor performance in cognitive behaviors [62]. It was necessary to arouse cortical arousal and change brain functions if smokers want to enhance performance in some cognitive behaviors. Only by acute cigarette smoking or injecting nicotine can smokers reduce negative attentional bias and increase cortical arousal though the neuro-chemically ascending cholinergic and noradrenergic projection of nicotine [4], [18], [40], [63]–[65]. Cortical arousal may also involve neuronal activation through nicotinic cholinergic receptors [22] or through the modulation of glutamate or GABA, dopamine neurotransmission, or MAO inhibitors [22], [62]. After nicotine administration, smokers could improve the performance in some cognitive behaviors, such as attention [18] and memory tasks [10].

One limitation of this study is that smoker and nonsmoker participants were treated differently: the smoker group was exposed to cigarettes whereas the nonsmoker group were not. The observed differences in smokers after acute cigarette smoking could therefore have been caused by psychological factors rather than the cigarettes. Thus, further research which could involve exposing nonsmoker groups to cigarettes or placebos is needed to confirm our results. Moreover, the verification of the SOA condition was based on a self-report rather than a confirmatory biological measure (e.g., breath carbon monoxide level). Furthermore, we were unable to determine the effect of acute cigarette smoking on different lengths of abstinence.

In conclusion, a novel GBC method was applied to investigate the influence of acute cigarette smoking on brain functions. The results indicated that GBC was higher in the insula and superior frontal gyrus in smokers under the SOA condition. Critically, lower GBC in the DMN was observed after acute cigarette smoking, which is associated with goal-directed cognitive performance. Furthermore, brain regions with structural changes partially overlapped with the affected hubs. In sum, this study may help elucidate the DMN regions that play an important role in smokers relapsing after a short period of time.
